# Work stress prevention needs of employees and supervisors

**DOI:** 10.1186/s12889-018-5535-1

**Published:** 2018-05-21

**Authors:** Bo M. Havermans, Evelien P. M. Brouwers, Rianne J. A. Hoek, Johannes R. Anema, Allard J. van der Beek, Cécile R. L. Boot

**Affiliations:** 1 Department of Public and Occupational Health, Amsterdam Public Health Research Institute, VU University Medical Center, PO box 7057, 1007 Amsterdam, MB The Netherlands; 2 Body@Work, Research Center Physical Activity, Work and Health, TNO-VU University Medical Center, Amsterdam, The Netherlands; 3 School of Social and Behavioural Sciences, Tranzo, Tilburg University, Tilburg, The Netherlands

**Keywords:** Work stress, Prevention, Intervention, Employee perspective, Needs, Implementation

## Abstract

**Background:**

Work stress prevention can reduce health risks for individuals, as well as organisational and societal costs. The success of work stress interventions depends on proper implementation. Failure to take into account the needs of employees and supervisors can hinder intervention implementation. This study aimed to explore employee and supervisor needs regarding organisational work stress prevention.

**Methods:**

Semi-structured telephone interviews were conducted with employees (*n* = 7) and supervisors (*n* = 8) from different sectors, such as the finance, health care, and services industry. The interviews focused on respondents’ needs regarding the prevention of work stress within an organisational setting. Performing thematic analysis, topics and themes were extracted from the verbatim transcribed interviews using Atlas.ti.

**Results:**

Both employees and supervisors reported a need for: 1) communication about work stress, 2) attention for determinants of work stress, 3) supportive circumstances (prerequisites) for work stress prevention, 4) involvement of various stakeholders in work stress prevention, and 5) availability of work stress prevention measures. Both employees and supervisors expressed the need for supervisors to communicate about work stress. Employees and supervisors reported similar psychosocial work factors that should be targeted for prevention (e.g., social support and autonomy). There was greater variety in the sub-themes within communication about work stress and supportive circumstances for work stress prevention in supervisor responses, and greater variety in the sub-themes within availability of work stress prevention measures in employee responses.

**Conclusions:**

Both employees and supervisors were explicit about who should take part in communication about work stress, what prerequisites for work stress prevention should exist, and which stakeholders should be involved. These results can inform work stress prevention practice, supporting selection and implementation of interventions.

**Trial registration:**

This study was registered in the Netherlands National Trial Register, trial code: NTR5527.

## Background

Work stress is associated with physical and mental health risks [1, 2]. In addition, it poses a financial burden to organisations and society at large, caused by productivity loss due to sickness absence, work disability benefits, and health care costs [3, 4]. If work stress could be prevented or reduced, its detrimental consequences could also be curtailed.

Prevention of work stress is supported by the availability of effective organisational stress management interventions (SMIs), and successful implementation of these interventions. Effective interventions do exist [5, 6], but they are not often used by organisations [7]. When SMIs are deployed, their usefulness depends greatly on proper implementation. Implementation is the extent to which the intervention is carried out as intended [8, 9]. The failure to implement an intervention properly, reduces its chances of rendering the desired effect [8, 10, 11].

Failure to take into account the needs of employees and supervisors can drastically hinder implementation of SMIs, because they are central stakeholders. Intervention development draws from research on work stress determinants, many of which are psychosocial work factors, as experienced by employees and supervisors [12, 13]. In widely used, hands-on approaches, such as participatory interventions [14] and intervention mapping [15], employees and supervisors work together with other stakeholders to select determinants of work stress to intervene upon. Employees and supervisors provide a valuable perspective, because they experience the current challenges in the organisation on a day-to-day basis. Reviews show that employees and their supervisors are often the main target population of SMIs [5, 6].

Despite awareness of their importance, employee and supervisor needs in work stress prevention are often not met. In a study by Aust and colleagues [16], employees withheld their participation in the intervention, because they felt it focused too much on leadership development, and too little on their perceived need for employee involvement. Dahl-Jorgensen and Saksvik [17] found that supervisors who were sceptical or negative towards the intervention restricted the time employees could spend on formulating prevention needs, thereby preventing implementation of a critical part of the intervention. Biron and colleagues [18] found that supervisors failed to use a stress risk assessment tool because they felt no need for stress prevention in the first place.

A better understanding of the organisational work stress prevention needs of employees and supervisors can promote the development, selection and implementation of SMIs. Therefore, this study aimed to explore employee and supervisor needs regarding organisational work stress prevention.

## Methods

### Design and procedure

A total of 15 semi-structured telephone interviews was conducted with employees (*n* = 7) and supervisors (*n* = 8), between January and March 2016. Employees were defined as non-managerial workers. Supervisors were defined as the management level that is just above the non-managerial employees. The interview focused on respondents’ experiences with work stress within the context of their organisation, and on their needs regarding the prevention of work stress within that organisational context. Respondents were at work or at home at the time of the interviews. They were asked to sit alone during the interview, in a place in which they could speak freely.

Respondents were primarily recruited through the network of the authors. Recruitment was done face-to-face, by telephone, and/or by email. Purposive sampling [19] was used to select respondents. This purposive sampling was aimed at selecting a diverse sample with regard to key criteria gender, age, educational level, and profession. To be eligible for participation, respondents had to be 18 years of age or older, and had to perform paid work. To maximise diversity in the study sample, the researchers made sure to select respondents who worked in different sectors (e.g., finance, health care), and held different positions (e.g., caregiver, fitness instructor, occupational physician). Some of the potential respondents were contacted directly, while others were recruited by their manager. Reasons for non-participation were: complexity of the subject, feeling ill-equipped to talk about the subject, and lack of interest in the subject. Four people who were scheduled to participate dropped out (*n* = 3) or were excluded (*n* = 1). The reason for exclusion was that the interviewer knew the possible respondent personally.

Interviews were conducted by telephone. First, the study was introduced to the respondents through an information folder, which was distributed to them by mail. Beforehand, respondents were asked to fill out an informed consent form. If the respondents had no further questions, the interview was started. Respondents received a gift coupon of ten Euros for their participation. The study has been assessed by the Medical Ethical Committee of VU University Medical Center, Amsterdam, The Netherlands.

### Interview protocol and interviewer

Different protocols were developed for employees and supervisors. The interview protocols consisted of a short introduction, and five questions (see Appendix), and were developed using existing qualitative research literature [19], as well as organisational stress prevention literature [8, 9]. The protocols were reviewed by all authors to assess face validity. For all questions, follow-up questions were formulated, to help the respondent elaborate on the subject. The follow-up questions were used only when necessary. The protocols were centred around the main theme: the respondent’s needs regarding work stress prevention. To make sure questions were clear to participants, a pilot test of three interviews (one supervisor, two employees) was conducted. This led to further clarification of items in the interview protocols. The first author (BMH) had had previous training in interviewing techniques and experience with interviewing, and conducted all interviews. The first author’s position was PhD Candidate, and he held a Master’s degree in Social Psychology. No relationship between the interviewer and the respondents existed prior to the study. No personal information was shared with respondents about the interviewer.

### Data collection

The telephone interviews were recorded. The interviewer made notes during the interview. On average, interview duration was 26 min. Saturation of the data was considered by the coders (BMH and RJAH) and the second author (EMPB). Saturation focused on between-interview occurrence of variation in answers to the questions [20, 21]. The interviews were conducted in two rounds to facilitate iteration, a reflexive process, in which collected data can be used to guide further data collection, or data analysis [22]. After the first round (*n* = 7), the recordings were inspected by both coders and the second author (EPMB), to see if adjustment of the interview protocols was necessary. This resulted in slight alterations of the protocols. For example, question 1 in both protocols was replaced with one of the follow-up questions (“What circumstances do you find important for the prevention or reduction of work stress?”). The altered protocols were used in the second (final) round (*n* = 8).

### Data analysis

The interview recordings were transcribed verbatim by a professional transcription agency. Transcripts were cross-checked for accuracy. The explorative objective of this study called for an approach that facilitates ‘bottom-up’ or inductive interpretation. Thematic analysis was considered an appropriate research methodology [23]. In thematic analysis, the researcher systematically works through the texts to identify topics, which are clustered in themes that have particular relevance to the research question [19]. The coders used a bottom-up, inductive coding style. Initial descriptive codes were applied to the transcripts, themes were developed at a later stage. Coding was done in four rounds, with coding of four transcripts in round 1, 2, and 4, and coding of three transcripts in round 3. During the coding rounds, reappearing themes (between transcripts) were isolated, which supported increasing, but gradual abstraction and summation of the data. In the coding process, the coders made sure that deviation from existing codes was facilitated, so that new codes could be added to the codes found earlier. They did this by looking for data units that were different from the reappearing themes already included in the coding structure [21]. After every round, the two coders first discussed their findings together, and presented their codes to the second author (EPMB) for further reflection (thematic analysis). The coding scheme was adapted after every round, so that new themes could be added, and code names could be consolidated. The COREQ-checklist was used in reporting this study [24]. Data management, coding and analysis was conducted using ATLAS.ti, version 7.5.11.

## Results

### Participants

The study sample consisted of eight females and seven males. Their age ranged from 29 to 63 years. Different sectors were represented in the study sample. Respondents worked in the finance (*n* = 7), health care (*n* = 5), and services sector (*n* = 3). Respondents had completed intermediate vocational education (n = 3), higher vocational education (*n* = 7), and university education (*n* = 5).

### Main themes

Themes and sub-themes from employees and supervisors are presented in Table 1. A five-theme structure was developed from the codes, which fitted both employee and supervisor responses. These main themes were 1) communication about work stress, 2) attention for determinants of work stress, 3) supportive circumstances for work stress prevention, 4) involvement of stakeholders in work stress prevention, and 5) availability of work stress prevention measures. All themes had sub-themes that represented different aspects of the main themes.

**Table 1:** Main Themes and Sub-Themes of Employee and Supervisor Work Stress Prevention Needs

Main themes	Sub-themes Employees	Sub-themes Supervisors
*Communication about work stress*	1. Supervisor initiates communication about work stress	1. Supervisor communicates about work stress
	2. Employee feels safe to talk about work stress	2. Employee feels safe to talk about work stress
		3. Employee indicates to supervisor if they experience stress
		4. Facilitation of communication between employee and (higher) management
*Attention for determinants of work stress*	1. Job demands	1. Job demands
	2. Co-worker support	2. Co-worker support
	3. Supervisor support	3. Supervisor support
	4. Autonomy/control	4. Autonomy/control
	5. Clarity about work tasks and expectations	
*Supportive circumstances for work stress prevention*	1. Supportive organisational culture	1. Supportive organisational culture
	2. Sufficient budget	2. Sufficient budget
		3. Organisation supports supervisor
		4. Employees’ readiness to change
*Involvement of stakeholders in work stress prevention*	1. External stakeholders	1. External stakeholders
	2. Internal stakeholders	2. Internal stakeholders
	3. Higher management/CEO	3. Higher management/CEO
	4. Supervisor	
*Work stress prevention measures*	1. Organisational measures	1. Organisational measures
	2. Competencies training	2. Cooperation between employee and supervisor
	3. Employee initiated action	
	4. Team meetings (including supervisor) about work stress	
	5. Exercise	

### Communication about work stress

A need for communication about work stress was expressed by both the employees and the supervisors. They both felt that the supervisor should communicate about work stress with the employees. Employees indicated that the supervisor should routinely initiate communication about work stress in an individual setting or in a team setting, with topics ranging from individual work stress experiences to reporting on topics relevant to work stress in employee satisfaction surveys. This way, the supervisor would be aware of work stress challenges that employees experience in their daily work:

*“[…] that my supervisor is aware of what their employee is working on, and regularly starts a conversation with them […]” (Respondent 3, employee)*

Supervisors also felt that they should communicate about work stress. However, they were less clear than employees about who would be responsible for initiating the conversation between supervisor and employee. Supervisors felt it was their responsibility to listen closely to employees, and to engage them in conversation about work stress:

*“[…] supervisors can reduce stress in employees by communicating, being clear and open towards employees […]” (Respondent 1, supervisor)*

However, both employees and supervisors expressed a need for employees to feel safe to talk about work stress. Both employees and supervisors reported the lack of employees feeling safe to talk about work stress:

*“If I would have the feeling that I could come to them [the organisation]. […] They [the organisation] only look at the numbers […] so I imagine that if I do something wrong, I am afraid I will be fired. It is a culture of fear.” (Respondent 13, employee)*

*“[…] that employees can express how they feel, even though it is very hard sometimes, and they do not feel safe to do so.” (Respondent 12, supervisor)*

Communication about work stress might be problematic if employees do not feel safe to talk about it because they are afraid of repercussions. Perhaps, this is why supervisors said that they had the need for employees to indicate to the supervisor if they experienced work stress, giving responsibility for communication about work stress (at least partly) back to employees. In multiple instances, supervisors reported that they could not help employees if they kept experiences of work stress to themselves:

*“I think the employee should clearly communicate about what is bothering him.” (Respondent 4, supervisor)*

*“I want employees to indicate they have work stress before it has gone on too long, so we can give them a few days off, rather than having them be on sick leave for longer periods of time” (Respondent 2, supervisor)*

Finally, supervisors expressed the need for communication about work stress between employees and (higher) management to be facilitated (e.g., by stimulating employees to bring ideas to management, or by management visiting the work floor more often). According to supervisors, this could lead to increased awareness of employees’ experiences on the one hand, and increased transparency of higher management about developments within the organisation on the other hand. Except for facilitation of communication between employees and (higher) management, all communication sub-themes were about interactions between employee and supervisor. For the most part, they seem to look at each other for communication about work stress.

### Attention for determinants of work stress

Respondents reported a need for attention for determinants of work stress. These determinants represented aspects of the work and the work environment that employees and supervisors felt should be targeted for work stress prevention. Both employees and supervisors indicated that they wanted attention for job demands:

*“There should be more attention for work tasks, making sure that demands are not too high for employees” (Respondent 7, employee)*

*“[…] that it will benefit everyone, if employees experience a balance in workload, job demands.” (Respondent 9, supervisor)*

Additionally, supervisors indicated that they experienced difficulty managing job demands, because it depended on factors that they could not control. Reported reasons for high job demands were: variability in production volume and personnel shortage:

*“What I noticed was that we had insufficient budget for personnel, and that we needed more people than we had available” (Respondent 8, supervisor)*

Employees and supervisors reported a need for attention for co-worker support. They felt that it was important to be able to depend on co-workers when there is much work to be done:

*“Being able to depend on your co-workers, well, […] that would contribute in a positive way.” (Respondent 11, employee)*

*“When one team has a lot of work to do and another team does not, the less busy team can help the busy team out.” (Respondent 8, supervisor)*

Both employees and supervisors indicated a need for attention for supervisor support. They stressed importance of help from supervisors with the work, and acknowledgement from supervisors and management for their work:

*“When employees indicate that there are difficulties with work tasks, the supervisor should help resolve those issues” (Respondent 3, employee)*

*“I think that a pat on the back [from the supervisor] works much better than reprimanding [the employee]” (Respondent 11, employee)*

*“It is all about looking at the dynamic and acknowledging every employee, so that everybody feels they are important.” (Respondent 12, supervisor)*

Another need reported by employees and supervisors was attention for autonomy/control. Employees emphasised a need for control over when they perform their working tasks, and over rostering and work days. Supervisors indicated a need for employees to have more control over their work schedule, but also expressed that this was sometimes difficult to achieve, suggesting that they did not always have the resources or skills necessary:

*“I want to be able to determine at what time I perform my work tasks” (Respondent 3, employee)*

*“An obstacle is that employees have too little influence, for example, they have too little influence on the work schedule. I have been thinking about this for a year now, but it is very hard to fix.” (Respondent 5, supervisor)*

Finally, employees indicated a need for more clarity about work tasks and expectations. Supervisors did not report this need. According to employees, clarity about work tasks and expectations would ensure that employees know what is expected of them. In addition, it would promote planning of work and accountability:

*“I would like to have more information about what has to happen [in the near future]. If I don’t have that information, I have to do everything all at once at the last moment.” (Respondent 14, employee)*

### Supportive circumstances for work stress prevention

This theme contains the need that employees and supervisors expressed for circumstances that would be supportive to work stress prevention. These circumstances could be interpreted as prerequisites for more active work stress prevention (such as an intervention), and as situations that respondents indicated they needed in order to prevent them from experiencing work stress.

A shared need of employees and supervisors was a supportive organisational culture. Supervisors were explicit about this being a responsibility of the whole organisation, but especially for higher management. The supportive organisational culture was described as a feeling of unity throughout the organisation, constructive criticism, a focus on people instead of numbers or money, and the ability to put things into perspective:

*“That is the most important thing, having that positive organisational culture, celebrate successes and making sure that you have a laugh every once in a while.” (Respondent 13, employee)*

*“I think there is a need for the entire organisation to understand that we are not working with employees and customers, but with people, […] that will create much more unity” (Respondent 1, supervisor)*

Both employees and supervisors reported a need for sufficient budget to be made available by the organisation for work stress prevention.

Supervisors reported two additional supportive circumstances for work stress prevention. Firstly, they indicated a need for organisational support. Specifically, they reported a need for organisational support for their managerial decisions and for their management style (also with regard to work stress prevention):

*“The organisation I work for gives me the opportunity to help balance [employees’] work and private life and that helps me to limit work stress” (Respondent 4, supervisor)*

Secondly, supervisors indicated that in order to prevent work stress, they needed employees to be ‘ready for change’. This individual readiness for change was described as the awareness and acknowledgement of work stress being a problem, willingness to participate in (and not resisting) the prevention process. A situation was described in which the employee does not seem to acknowledge work stress; in another situation that was described, the employee acknowledged the presence of work stress, but did not want to use solutions provided:

*“By not giving any signal of work stress, probably because they did not acknowledge it being there, the employee had a definite role in being burnt out. So it is necessary that the employee is aware of their capacity to work and the circumstances that can lead to sickness absence.” (Respondent 9, supervisor)*

*“He [the employee] does not really respond to the solutions that I have provided, like a stress management training, or scheduling skills, and I find that worrisome.” (Respondent 10, supervisor)*

### Involvement of stakeholders in work stress prevention

Employees and supervisors indicated a need for several different stakeholders to be involved in work stress prevention. A differentiation can be made between the stakeholders with respect to their being part of the organisation (i.e. internal stakeholders) or external to the organisation (i.e. external stakeholders). Examples of external stakeholders that respondents mentioned were companies that provide work stress management trainings, and occupational health professionals who can help with job redesign. Examples of internal stakeholders that employees and supervisors mentioned were: the job coach, human resources, and the occupational physician. Employees specifically mentioned that they wanted the occupational physician to be involved in an earlier stage of work stress prevention (i.e. primary or secondary prevention). Supervisors specifically mentioned the occupational physician and human resources:

*“Only when the problem is already very severe and you cannot work anymore, only then the occupational physician is involved.” (Respondent 7, employee)*

*“I have approached human resources to ask what their policy was, what they had in mind [regarding work stress prevention]” (Respondent 1, supervisor)*

*“[The occupational physician] should preferably be involved before the employee is sick, so the employee indicates that things are not going well, and then we decide that contacting the occupational physician is a good idea.” (Respondent 2, supervisor)*

Examples of external stakeholders that were mentioned by employees and supervisors were occupational health professionals, trainers, and physiotherapists.

Because of substantial prominence (being mentioned by almost all employees and several supervisors), higher management/CEOs are mentioned in a separate sub-theme, instead of being grouped with the internal stakeholders described earlier. Employees predominantly expressed an absence of sufficient involvement of higher management and/or the CEO in work stress prevention, whereas supervisor reports were more mixed:

*“Top management should pay a lot more attention to stress and that their responsibility is to make sure that employees can work in health and safety.” (Respondent 13, employee)*

*“Luckily, our upper management is involved with the individuals working in the organisation, […] but in general, it is all about reports and statistics.” (Respondent 5, supervisor)*

Employees reported a need for the supervisor to be involved in work stress prevention. Involvement of supervisors was characterised as: guiding the work stress prevention process, providing insight into work stress prevention options and availability of tools, helping to find sources of work stress and resources for healthy work:

*“What I expect from my supervisor is that they help to find out where the work stress is coming from if the employee is unable to pinpoint it […], and take action if there is prolonged work stress.” (Respondent 14, employee)*

### Work stress prevention measures

Employees and supervisors expressed a need for work stress prevention measures. They shared a need for organisational measures, such as hiring more personnel to reduce workload, introducing protocols, better planning of work tasks/shifts:

*“A better planning […] if we would discuss that more regularly, we could let more things run smoothly, and remove stressors that way.” (Respondent 15, employee)*

*“Good planning and accurate prediction of what workload we should expect and having a buffer for calamities.” (Respondent 8, supervisor)*

Other than organisational measures, employees and supervisors did not share needs for measures. Employees indicated a need for exercise measures, such as going for walks or participating in sports. They also reported a need for employee-initiated action, or measures that they could use without depending on other stakeholders in the organisation, such as relaxation, exercises, and taking breaks regularly. Employees also expressed a need for team meetings with the supervisor that focus on work stress:

*“It’s not like we have a meeting focusing on work stress alongside the employee satisfaction surveys, that look at the individual level, so we can make arrangements [for work stress prevention]” (Respondent 6, employee)*

Lastly, employees expressed a need for competencies training aimed at coping with stress, setting boundaries, and dealing with changes:

*“We do not have the knowledge and skills to switch from one [computer] system to the other in a day, for that, we need help, in a training.” (Respondent 14, employee)*

Supervisors reported a need for measures that increase cooperation between the employee and the supervisor. Examples of such measures were facilitating asking of questions during team meetings, and working together to optimise the work atmosphere.

## Discussion

A diverse set of needs regarding work stress prevention was reported by the employees and supervisors who participated in the current study, illustrating that work stress prevention is a multi-faceted and complex challenge. At the same time, themes could be recognised from the responses, and commonalities between employees and supervisors could be found.

Both employees and supervisors reported a need for communication about work stress. Communication has been argued to facilitate awareness raising [25] and selection of SMIs [8]. It is represented in models for intervention evaluation [9], and has been found to be relevant in the SMI implementation process [26]. Employees were hesitant to initiate communication about work stress because they feared this would affect their position. This notion is illustrated strikingly by the second quote about a “culture of fear” under paragraph “Communication about work stress” in the results section. Moreover, previous research suggests that job insecurity has increased in the past decades [27]. As supervisors also expressed the need for employees to feel safe to talk about work stress and the need for employees to indicate if they experienced stress, an impasse becomes apparent. Supervisors can make less informed decisions about work stress prevention if employees give no indication of work stress, and employees are limited to more individual resources for work stress prevention that could strengthen their position and employability. Other barriers to communication about work stress may exist. For instance, in an assessment of factors relevant to stress and mental ill-health prevention, Moll [28] reported that stigma regarding mental health issues hindered communication about work stress. Stigma was one of multiple barriers to communication about mental health issues, creating a “web of silence”. Another example of a barrier to communication about mental health issues such as work stress is that supervisors might have insufficient knowledge about these issues [29]. Open communication about mental health issues within the context of the workplace can be very difficult [30]. However, the findings of the current study show that employees and supervisors did want a dialogue about work stress. Employees and supervisors reported this need independently from each other, as they did not belong to the same department, and in most cases not even to the same organisation. In addition, supervisors wanted higher management to participate in this dialogue as well.

Great overlap existed between employees and supervisors concerning the need for attention for determinants of work stress. This overlap existed regardless of the fact that the employees and the supervisors were not from the same departments (and in most cases not from the same organisations). They both reported the need for attention for job demands, co-worker and supervisor support, and autonomy/control. These psychosocial work factors are best represented in the job demands-control (support) model [12, 31], and represent fundamental psychosocial challenges in maintaining a healthy workforce. An exception was the need for clarity about work tasks and expectations, which was reported by employees only. Lack of clarity of work tasks has been found to be associated with psychological distress [32]. Supervisors could prevent work stress in employees by helping them to express their expectations more clearly.

A need for supportive circumstances for work stress prevention was shared by employees and supervisors, but only for sub-themes “supportive organisational culture” and “sufficient budget”. Supervisors had a more varied need for supportive circumstances, at the level of the organisation (organisational support for supervisor) and at the level of the employee (readiness for change). The importance of organisational support and readiness for change has been stressed in existing literature [8, 9, 33]. Like the employees, supervisors reported a need for support regarding work stress prevention from their superiors in the organisation. Supervisors may sometimes feel trapped in between employees not willing to acknowledge work stress and participate in possible solutions on the one hand, and management unwilling to provide resources and support on the other hand. It serves as a reminder that different stakeholders (e.g., management/CEOs, social partners) should be involved for work stress prevention to succeed, as has been recommended in previous publications [8, 9, 25, 34].

Within the main theme about the involvement of stakeholders in work stress prevention, employees and supervisors contended that the occupational physician (internal stakeholder) should be involved in the work stress prevention process. Until recently in Dutch health care, the occupational physician was mainly involved in tertiary prevention, or making sure that people return to work after sickness absence. In June 2017, new legislation from the Dutch Ministry of Social Affairs and Employment went into effect stating that occupational physicians should also advise organisations about work stress prevention, and not focus primarily on reduction of sickness absence [35]. Hopefully, this new legislation may pull the occupational physician more to the forefront of work stress prevention, possibly advising to employ professionals (e.g., trainers) or to deploy measures that can help the organisation and its employees to deal with work stress, which was another need employees and supervisors agreed upon. Even though both employees and supervisors reported a need for higher management/CEOs to be involved in work stress prevention, the need was much more prominent in employees. Research indicates that the extent to which employees feel that senior management finds a good psychosocial work environment to be important (i.e. psychosocial safety climate) [36] is negatively associated with work stress and a with psychological health problems [37, 38]. The importance of visibility and involvement of higher management in work stress prevention is reconfirmed by the findings in the current study.

Employees and supervisors reported a need for organisational work stress prevention measures. It was the only sub-theme that they shared in the main theme “work stress prevention measures”. An argument for deployment of organisational-level interventions is the “hierarchy of controls”, which is a general principle in occupational health that states that the further upstream an intervention is, the greater the effect of the intervention will be [39]. It should be noted that the need expressed by employees for team meetings (including supervisor) about work stress would probably constitute cooperation between employee and supervisor. This indicates that there may have been more shared needs within the main theme “work stress prevention measures” beyond the need for organisational work stress measures. Involvement of different stakeholders and activities at different levels is in line with recommendations made for effective stress management and mental health promotion in employees [40, 41]. In this regard, the needs reported in the current study suggest that an integrated approach to work stress prevention might be necessary. The fact that employees indicated a greater variety of needs for work stress prevention measures may be because they experience the challenges first-hand. This might give them better insight into which measures are necessary to combat work stress. The sub-themes reported by employees indicated that they felt at least in part responsible for work stress prevention: they indicated a need to learn to deal with work stress by taking part in competencies training, a need for employee initiated action, and a need for physical exercise. Still, finding appropriate measures seems to be a challenge for organisations [7], so greater insight of different stakeholders into the relevant options might be beneficial.

### Strengths and limitations

A strength of this study was its selection of a diverse sample of employees and supervisors. The fact that employees and supervisors did not belong to the same departments or organisations may have encouraged respondents to freely speak about their needs, without expectations, and without fear for repercussions. For purposeful sampling, we used our professional network. This rendered a heterogeneous group, but because we recruited from our own network, there was a chance of bias. To prevent this bias, personal acquaintances of the interviewer were excluded. The qualitative approach facilitated spontaneous conceptualisation of new concepts relevant to work stress prevention. Moreover, the analysis phase was extensive and carefully planned, and was conducted by two authors (BMH and RJAH). Previous literature suggests a minimum of six to twelve interviews to reach saturation [42]. This criterion was met in the current study, with 15 interviews conducted. Moreover, commonalities and recurring themes were found (and agreed upon by coders BMH and RJAH, and second author EPMB) within groups in the diverse sample, indicating that saturation was reached, and that this study constitutes a viable exploration of work stress prevention needs.

The study also has limitations that should be taken into account. The main limitation of this research relates to generalizability. For example, our study contained no respondents of the lowest education level participated, and was conducted in the context of the Dutch social security system, which differs from that of other countries. However, generalizability was not the purpose of the study. As with other qualitative studies, the aim was to provide insight into a complex challenge of which more knowledge is urgently needed. The interview study approach, and diversity of our sampling, ensured inclusion of perspectives and experiences from a wide range of participants with different characteristics. This provides a multifaceted picture that would not have been available using surveys or other observational methods. Finally, no feedback on the findings was asked from respondents.

The fact that the interviews were conducted by telephone presents methodological strengths as well as limitations. On the one hand, respondents had more anonymity talking about the sensitive topic of work stress, possibly prompting less social desirable answers. On the other hand, more sensitive parts may have been picked up less by the interviewer, due to reduced registration of non-verbal communication. Sturges and Hanrahan compared face-to-face and telephone interviewing modes, and concluded that no significant data quality differences existed between the two modes [43]. This finding strengthens the position that for the current study, telephone interviews were appropriate.

### Implications for research, practice, and policy

It should be noted that the interviews in this study focused on work stress prevention in employees, and not necessarily on supervisors’ own personal needs. Supervisors did express the need for support from their own superiors, but to get a more complete account of work stress challenges that are specific to supervisors and other important stakeholders, different questions should be asked, and in some cases, different stakeholder should be approached. In order to find more generalizable work stress prevention needs, this study could be carried out using a larger sample that is more diverse with respect to educational level, and that has more distinct levels of work stress. Findings of such research may assist translation of work stress prevention theory into practice, contributing to bridging the gap between work stress models and intervention implementation. Fortunately, exploration of work stress prevention needs plays a role in widely used frameworks for (occupational) health promotion, such as intervention mapping [15, 44]. Still, intervention mapping is usually carried out within a single organisation, providing a view of occupational health challenges that is specific to that organisation. Population-based exploration may provide insights that can advance prevention practice in different ways than exploration within an organisation. Policy makers and research funding agencies could stimulate qualitative research of work stress prevention.

## Conclusions

The findings of this study indicate that employees and supervisors have multifaceted work stress prevention needs. Despite the fact that employees and supervisors did not work at the same departments/organisations, their responses pointed at similar needs. At the same time, distinct differences existed between the two groups. As employees and supervisors are both key stakeholders in work stress prevention, mapping their needs could facilitate intervention implementation.

## Appendix

**Table 2:** Interview Questions for Employees and Supervisors

Employees Introduction: This interview is about work stress. During the interview, I would like to talk about two things: your own experience with work stress and work stress prevention. By work stress we do not mean regular excitement that people might find agreeable, but actual tension or stress that feels uncomfortable, and is related to work. First, I would like to ask you some questions about your own experience with work stress. Then, I would like to ask you some questions about what you think would be necessary within your organisation to prevent or reduce work stress. The interview will take approximately 30 min. Do you have any questions? Then let us begin: 1. Can the work stress you (might) experience be reduced or prevented? Do you want that? What is required to achieve this? What circumstances do you find important for the prevention or reduction of work stress? What could hinder prevention or reduction of work stress? 2. What should happen within your organisation to prevent or reduce work stress? Do you think this is necessary? What does it take to make this happen? 3. What role do you play in preventing or reducing work stress? Would you be willing to play a role? What would facilitate this? 4. What other parties can support prevention or reduction of work stress? What can your supervisor do? What can your company physician do? What can your colleagues do? Are there any others that can support work stress reduction or prevention? 5. What advice would you give to your supervisor regarding the work stress prevention needs of employees? What advice would you give to your organisation? Are there others you would like to give advice? Supervisors Introduction: This interview is about work stress. During the interview, I would like to talk about two things: your experience with work stress in the employees that you supervise and work stress prevention. By work stress we do not mean regular excitement that people might find agreeable, but actual tension or stress that feels uncomfortable, and is related to work. First, I would like to ask you some questions about stress in your workplace. These question are about work stress in your employees specifically. Then, I would like to ask you some questions about what you think would be necessary within your organisation to prevent or reduce work stress. The interview will take approximately 30 min. Do you have any questions? Then let us begin: 1. Can the work stress that employees that you supervise (might) experience be reduced or prevented? Do you think this is necessary? Do you want this? What is required to achieve this? What circumstances do you find important for the prevention or reduction of work stress? What could hinder prevention or reduction of work stress? 2. What role do you play in prevention or reducing work stress? Would you be willing to play a role? What would you need to facilitate this? 3. What other parties can support prevention or reduction of work stress? What can your company physician do? What can your colleagues do? What can employees do? Are there any others that can support work stress reduction or prevention? 4. How would you describe the organisational climate when it comes to work stress? Is it possible to discuss work stress? What can your supervisor do to prevent or reduce work stress? How do influential persons within your organisation deal with indications of work stress? 5. What advice would you give to your organisation regarding the work stress prevention needs of supervisors? What advice would you give to your colleagues? Are there others you would like to give advice?	

## Abbreviations

SMI: Stress Management Intervention

## References

[1] Ganster DC, Rosen CC (2013). Work stress and employee health: a multidisciplinary review. J Manage.

[2] Steptoe A, Kivimaki M (2013). Stress and cardiovascular disease: an update on current knowledge. Annu Rev Public Health.

[3] Henderson M, Glozier N, Elliott KH (2005). Long term sickness absence: is caused by common conditions and needs managing. Brit Med J.

[4] Hassard J, Teoh K, Cox T, Dewe P, Cosmar M, Gründler R (2014). Calculating the costs of work-related stress and psychosocial risks.

[5] van der Klink JJ, Blonk RW, Schene AH, van Dijk FJ (2001). The benefits of interventions for work-related stress. Am J Public Health.

[6] LaMontagne AD, Keegel T, Vallance D (2007). Protecting and promoting mental health in the workplace: developing a systems approach to job stress. Health Promot J Austr.

[7] Westgaard RH, Winkel J (2011). Occupational musculoskeletal and mental health: significance of rationalization and opportunities to create sustainable production systems: a systematic review. Appl Ergon.

[8] Nytro K, Saksvik PO, Mikkelsen A, Bohle P, Quinlan M (2000). An appraisal of key factors in the implementation of occupational stress interventions. Work Stress.

[9] Nielsen K, Randall R (2013). Opening the black box: presenting a model for evaluating organizational-level interventions. Eur J Work Org Psychol.

[10] Murta SG, Sanderson K, Oldenburg B (2007). Process evaluation in occupational stress management programs: a systematic review. Am J Health Promotion.

[11] Nielsen K, Randall R (2012). The importance of employee participation and perceptions of changes in procedures in a teamworking intervention. Work Stress.

[12] Johnson JV, Hall EM (1988). Job strain, work place social support, and cardiovascular disease: a cross-sectional study of a random sample of the Swedish working population. Am J Pub Health.

[13] Demerouti E, Bakker AB, Nachreiner F, Schaufeli WB (2001). The job demands-resources model of burnout. J Appl Psychol.

[14] McVicar A, Munn-Giddings C, Seebohm P (2013). Workplace stress interventions using participatory action research designs. Int J Workplace Health Manage.

[15] Bartholomew LK, Parcel GS, Kok G (1998). Intervention mapping: a process for developing theory and evidence-based health education programs. Health Educ Behav.

[16] Aust B, Rugulies R, Finken A, Jensen C (2010). When workplace interventions lead to negative effects: learning from failures. Scand J Pub Health.

[17] Dahl-Jorgensen C, Saksvik PO (2005). The impact of two organizatoinal interventions on the health of service sector workers. Int J Health Serv.

[18] Biron C, Gatrell C, Cooper CL (2010). Autopsy of a failure: evaluating process and contextual issues in an organizational-level work stress intervention. Int J Stress Manage.

[19] Ritchie J, Lewis J, Nicholls CM, Ormston R: Qualitative research practice: A guide for social science students and researchers. SAGE Publications Ltd; 2013.

[20] Morse JM (1995). The significance of saturation. Qual Health Res.

[21] Bowen GA (2008). Naturalistic inquiry and the saturation concept: a research note. Qual Res.

[22] Srivastava P, Hopwood N (2009). A practical iterative framework for qualitative data analysis. Int J Qual Methods.

[23] Braun V, Clarke V (2006). Using thematic analysis in psychology. Qual Res Psychol.

[24] Tong A, Sainsbury P, Craig J (2007). Consolidated criteria for reporting qualitative research (COREQ): a 32-item checklist for interviews and focus groups. Int J Qual Health Care.

[25] Kompier MAJ, Marcelissen FHG (1995). Handboek werkstress: systematische aanpak voor de bedrijfspraktijk.

[26] Havermans BM, Schelvis RM, Boot CR, Brouwers EP, Anema JR, van der Beek AJ (2016). Process variables in organizational stress management intervention evaluation research: a systematic review. Scand J Work Environ Health.

[27] Landsbergis PA, Grzywacz JG, LaMontagne AD (2014). Work organization, job insecurity, and occupational health disparities. Am J Ind Med.

[28] Moll SE (2014). The web of silence: a qualitative case study of early intervention and support for healthcare workers with mental ill-health. BMC Public Health.

[29] Gayed A, Milligan-Saville JS, Nicholas J, Bryan BT, LaMontagne AD, Milner A, et al. Effectiveness of training workplace managers to understand and support the mental health needs of employees: a systematic review and meta-analysis. Occup Environ Med. 2018:1–9. 10.1136/oemed-2017-104789 29563195

[30] Corbiere M, Villotti P, Toth K, Waghorn G (2014). Disclosure of a mental disorder in the workplace and work accommodations: two factors associated with job tenure of people with severe mental disorders. L'Encephale.

[31] Karasek RA (1979). Job demands, job decision latitude, and mental strain: implications for job redesign. Adm Sci Q.

[32] Bliese PD, Castro CA (2000). Role clarity, work overload and organizational support: multilevel evidence of the importance of support. Work & Stress.

[33] McVicar A (2003). Workplace stress in nursing: a literature review. J Adv Nurs.

[34] Leka S, Jain A, Cox T, Kortum E (2011). The development of the European framework for psychosocial risk management: PRIMA-EF. J Occup Health.

[35] Ministry of Social Affairs and Employment. 1–1-2017. 20–6-2017. Ref Type: Online Source. https://www.arboportaal.nl/actueel/arbozorg/wijzigingen. Accessed 20 May 2017

[36] Hall GB, Dollard MF, Coward J (2010). Psychosocial safety climate: development of the PSC-12. Int J Stress Manage..

[37] Idris MA, Dollard MF, Coward J, Dormann C (2012). Psychosocial safety climate: conceptual distinctiveness and effect on job demands and worker psychological health. Saf Sci.

[38] Havermans BM, Boot CRL, Houtman ILD, Brouwers EPM, Anema JR, van der Beek AJ (2017). The role of autonomy and social support in the relation between psychosocial safety climate and stress in health care workers. BMC Pub Health.

[39] LaMontagne AD, Keegel T, Louie AM, Ostry A, Landsbergis PA (2007). A systematic review of the job-stress intervention evaluation literature, 1990-2005. Int J Occup Environ Health.

[40] Grawitch MJ, Ballard DW, Erb KR (2015). To be or not to be (stressed): the critical role of a psychologically healthy workplace in effective stress management. Stress Health.

[41] LaMontagne AD, Martin A, Page KM, Reavley NJ, Noblet AJ, Milner AJ (2014). Workplace mental health: developing an integrated intervention approach. BMC Psychiat.

[42] Guest G, Bunce A, Johnson L (2006). How many interviews are enough? An experiment with data saturation and variability. Field methods.

[43] Sturges JE, Hanrahan KJ (2004). Comparing telephone and face-to-face qualitative interviewing: a research note. Qual Res.

[44] Eldredge LKB, Markham CM, Kok G, Ruiter RA, Parcel GS: Planning health promotion programs: an intervention mapping approach. Wiley; 2016.

